# The impact of Enhanced Recovery after Surgery (ERAS) pathways with regard to perioperative outcome in patients with ovarian cancer

**DOI:** 10.1007/s00404-021-06339-6

**Published:** 2021-12-27

**Authors:** Susanne Reuter, Linn Woelber, Constantin C. Trepte, Daniel Perez, Antonia Zapf, Sinan Cevirme, Volkmar Mueller, Barbara Schmalfeldt, Anna Jaeger

**Affiliations:** 1 Department of Gynecology, Hamburg-Eppendorf University Medical Center, Martinistraße 52, 20246 Hamburg, Germany; 2 Department of Anaesthesiology, Hamburg-Eppendorf University Medical Center, Hamburg, Germany; 3 Department of General, Visceral and Thoracic Surgery, Hamburg-Eppendorf University Medical Center, Hamburg, Germany; 4 Institute of Medical Biometry and Epidemiology, University Medical Centre Hamburg-Eppendorf, Hamburg, Germany

**Keywords:** Enhanced Recovery after Surgery (ERAS), Perioperative outcome, Length of hospitalization/stay (LOS), Gynecologic oncology, Ovarian cancer

## Abstract

**Purpose:**

Major surgery for ovarian cancer is associated with significant morbidity. Recently, guidelines for perioperative care in gynecologic oncology with a structured “Enhanced Recovery after Surgery (ERAS)” program were presented. Our aim was to evaluate if implementation of ERAS reduces postoperative complications in patients undergoing extensive cytoreductive surgery for ovarian cancer.

**Methods:**

134 patients with ovarian cancer (FIGO I-IV) were included. 47 patients were prospectively studied after implementation of a mandatory ERAS protocol (ERAS group) and compared to 87 patients that were treated before implementation (pre-ERAS group). Primary endpoints of this study were the effects of the ERAS protocol on postoperative complications and length of stay in hospital.

**Results:**

Preoperative and surgical data were comparable in both groups. Only the POSSUM score was higher in the ERAS group (11.8% vs. 9.3%, *p* < 0.001), indicating a higher surgical risk in the ERAS group. Total number of postoperative complications (ERAS: 29.8% vs. pre-ERAS: 52.8%, *p* = 0.011), and length of hospital stay (ERAS: 11 (6–23) vs pre-ERAS: 13 (6–50) days; *p* < 0.001) differed significantly. A lower fraction of patients of the ERAS group (87.2%) needed postoperative admission to the ICU compared to the pre-ERAS group (97.7%), *p* = 0.022). Mortality within the ERAS group was 0% vs. 3.4% (*p* = 0.552) in the pre-ERAS group.

**Conclusion:**

The implementation of a mandatory ERAS protocol was associated with a lower rate of postoperative complications and a reduced length of stay in hospital. If ERAS has influence on long-term outcome needs to be further evaluated.

## Introduction

The Enhanced Recovery after Surgery (ERAS) “program has been developed based on the principle of fast track surgery in colorectal surgery [1–6]. It then has been adopted to many other patient populations [1, 7]. The main goal of ERAS, which is an empirically developed bundle of different interventions, is to reduce perioperative stress. Although not all of the interventions of ERAS are evidence based when assessed separately [8–11], the combination to a multimodal approach seems to improve outcome in terms of reducing perioperative complications, length of stay (LOS) in hospital and hospital re-admissions. Main components of ERAS are: atraumatic, i.e. laparoscopic surgical techniques, extended preoperative patient information and education, the avoidance of preoperative bowel preparation, decrease of preoperative fasting, preoperative metabolic optimization by carbohydrate loading, early postoperative enteral feeding, avoidance or early abandoning of wound drains and feeding tubes, improved peri- and postoperative pain therapy using epidural anesthesia and opioid sparing medication, and early mobilization [3–7]. The ERAS society published a first edition of ERAS guidelines for gynecological oncological surgery in 2016, which is very closely aligned to the guidelines for colorectal surgery [2]. However, epithelial ovarian cancer has the highest mortality of all gynecological tumors [12]. One reason is the fact that diagnosis often is made very late with already advanced tumor progression (FIGO stadium IIB and higher), frequently already associated with peritoneal carcinomatosis. Then, gold standard of therapy consists of the combination of complete cytoreductive surgery (debulking) and adjuvant platin-based systemic chemotherapy. The macroscopically complete cytoreduction is the most important factor of prognosis [13, 14]. Due to tumor dissemination within the whole abdomen, the surgical approach frequently requires complex multivisceral resections: this includes resection of all tumor localizations, radical hysterectomy and bilateral ovariectomy, infragastric omentectomy, and for FIGO stage I-II additionally paraaortic and pelvic radical lymphonodectomy. Further, frequently extensive deperitonealization of the pelvis, colonic rims and the diaphragm, as well as resections often the large and/or small intestines, in particular the deep anterior resection of the rectum, splenectomy, cholecystectomy, partial liver resections, and resections of all bulky lymph nodes are often necessary. These extensive surgical interventions normally do not allow laparoscopic techniques, and moreover, cause a massive surgical trauma with concomitant severe systemic inflammation. This also explains higher perioperative complication and mortality rates, the latter up 6% [15]. Another important prognostic factor is the time interval between surgery and initiation of adjuvant chemotherapy. Therefore, fast recovery after cytoreductive surgery is of high importance [16]. ERAS might therefore have beneficial consequences in particular in this group of patients. However, there is so far only sparse evidence on this. In 2018, we implemented a strict and mandatory ERAS protocol in the treatment pathways for ovarian cancer surgery in our department. We hypothesized that this implementation will reduce postoperative complications. We therefore prospectively collected data of all patients after implementation of the ERAS protocol, and compared this to a set of retrospectively assessed data of all patients from 2015 to 2017.

## Methods

### Study design

This study is an analysis of the prospectively collected data of patients scheduled for suspected or diagnosed ovarian cancer or recurrent ovarian cancer scheduled for cytoreductive surgery after implementation of an ERAS program in the year 2018 (ERAS group), and a comparison to retrospectively acquired data of a historical control group of patients treated with the same diagnosis between 2015 and 2017 (pre-ERAS group).

### Study participants and treatment strategies

ERAS Group: in this group all patients admitted to our department with suspected or diagnosed ovarian cancer and the indication of cytoreductive tumor surgery via laparotomy were included. Preoperative preparation, perioperative management, and postoperative treatment was standardized according to the published ERAS guidelines [1].

The single elements of this patient management, such as renunciation of bowel preparation, preoperative carbohydrate-loading, early mobilization, and principles of fluid- and pain therapy were obligatory settled in written standard operating procedures. Furthermore, all pre-, peri-, and postoperative data were systematically collected according to the requirements of the database of the International ERAS society. Additionally, values of hemoglobin, thrombocytes, and amount of ascites were assessed.

Pre-ERAS group: for this group data were acquired from patients who were treated with cytoreductive surgery because of the same oncological indication in our department between 2015 and 2017. All suitable patient charts of this period were screened in detail. Patients were included, if at least 80% of data, which is assessed in the ERAS database, was available.

### Statistical analysis

Data were analyzed using R, version 4.0.3 [17]. The two-sided type-one error is set to 5%. The two primary hypotheses regarding complications and length of hospital stay were ordered hierarchically. Therefore, no adjustment for multiplicity was necessary. All further analyses were exploratory and the *p* values were interpreted as descriptive measures.

Normally distributed variables are reported with mean and standard deviation and analyzed with a linear ANOVA. Variables with skewed distributions are reported with median and IQR (interquartile range) and were either transformed using the logarithm function and analyzed with a linear ANOVA where applicable or with Kruskal–Wallis’ rank sum test. Fisher’s exact test was used for categorical variables. Distribution assumptions were made based on histograms and measures of location. Results were considered to be significant if *p* < 0.05.

## Results

Acquisition of all data was approved by the Ethics Committee of the Medical Board Hamburg (PV190504). Informed consent was obtained from all patients.

Between January 1 2018, and December 31, 2019, 47 patients were prospectively included into the ERAS group. For the pre-ERAS cohort, we screened in total medical charts of 153 patients who underwent laparotomy for ovarian cancer in the years 2015–2017. Sixty-six patients were excluded, because of incompleteness of data sets according to the ERAS-database. Finally, 87 patients were analyzed.

### Demographic data and perioperative risk stratification

Data are given in Table 1. Although patients of the pre-ERAS group tended to be younger, both groups did not differ considerably regarding age or body mass index. Overall surgical risk stratification was assessed according to the preoperative risk score of the American Society of Anesthesiologists (ASA-score) [18] and the Physiological and Operative Severity Score for the enumeration of Mortality and morbidity (POSSUM) [19]; patients of the ERAS group had a higher POSSUM score, indicating a slightly higher perioperative risk for complications (pre-ERAS group: 9.3% vs. ERAS group: 11.8%; *p* < 0.001).

**Table 1:** Demographic patient data and perioperative risk stratification

Characteristics	Pre-ERAS group *n* = 87	ERAS group *n* = 47	Total *n* = 134	*P* value
Age, median (IQR)	60 (52–70.5)	65 (56.5–70)	60 (54–70.8)	0.105
BMI (kg/m^2^), median (IQR)	25.4 (22.2–28)	25.4 (22.2–30.5)	25.4 (22.2–29.2)	0.200
ASA (*n* %)				0.436
1	0 (0%)	0 (0%)	0 (0%)	
2	43 (49.4%)	23 (48.9%)	66 (49.3%)	
3	40 (46%)	24 (51.15)	64 (47.8%)	
4	4 (4.6%)	0 (0%)	4 (3.0%)	
POSSUM Score, (mean)	9.3% 9.434 ± 2.071	11.8% 11.880 ± 2.122	10.280 ± 2.386	*** < 0.001***
Hb (g/dl)	12.124 ± 1.390	12.400 ± 1.582	12.220 ± 1.459	0.302
Thrombocyte (/nl)	371.1 ± 123.69	350.3 ± 120.03	363.8 ± 122.37	0.350

*BMI* body mass index; *ASA* American Society of Anesthesiologists; *POSSUM* Score Physiologic and Operative Severity for en Umeration of Mortality and Morbidity; *Hb* haemoglobin; *ERAS* enhanced recovery after surgery; *SD* standard deviation

### Surgical and perioperative data

Data are summarized in Table 2. In the ERAS group, 85.1%, and in the pre-ERAS group 88.5% of patients had primary surgery. Interval surgery was performed in 14.9% in the ERAS group vs. 11.5% in the pre-ERAS group. Histopathological staging according the FIGO classification did not differ significantly between both groups. There were no relevant differences between both groups with regard to the extent of surgery of organ resection in the pelvis, and the lower, and the upper abdomen. However, in the ERAS group, systematic pelvic and aortic lymphonodectomy was performed to a lower degree (ERAS group: 31.9% vs pre-ERAS group: 64.4%; *p* < 0.001). Patients of the ERAS group received considerably fewer crystalloid fluids during surgery (3500 (2500–4500) vs. 4000 (3000–5000) ml *p* = 0.036). Other perioperative data did not reveal any relevant differences.

**Table 2:** Surgical and perioperative data

Characteristics	Pre-ERAS-group *n* = 87	ERAS-group *n* = 47	Total *n* = 134	*P* value
Indication for CRS (*n*. %)				
Primary	77 (88.5%)	40 (85.1%)	117 (87.2%)	0.156
Interval	10 (11.5%)	7 (14.9%)	17 (12.8%)	
				0.485
FIGO I-II	17 (19.5%)	8 (17.0%)	25 (18.7%)	
FIGO IIIA/B	12 (13.8%)	4 (8.5%)	16 (11.9%)	
FIGO IIIC	43 (49.4%)	28 (59.6%)	71 (53.0%)	
FIGO IVA	2 (2.3%)	3 (6.4%)	5 (3.7%)	
FIGO IVB	10 (11.5%)	2 (4.3%)	12 (9.0%)	
Recurrent	2 (2.3%)	2 (4.3%)	4 (3.0%)	
Unknown	1 (1.1%)	0 (0%)	1 (0.7%)	
Type of surgery (*n*. %)				
HE, BSE, Omentectomy	78 (89.7%)	44 (93.6%)	122 (91.0%)	0.540
Upper abdominal surgery (Splenectomy, Hepatectomy, small bowel resection, complete diaphragma stripping, etc.)	80 (92.0%)	37 (78.7%)	117 (87.3%)	0.054
Systemtatic LNE paraaortal, pelvin	56 (64.4%)	15 (31.9%)	71 (53.0%)	** < *0.001***
Bowel anastomosis	47 (54%)	27 (57.4%)	74 (55.2%)	0.720
Operating time, h	5.06 ± 1.43	4.63 ± 1.31	4.91 ± 1.39	0.093
Intraoperative blood loss, ml, median, (IQR)	800 (500–6500)	800 (400–1200)	800 (400–1200)	0.645
Residual disease (*n*. %)				
None	47 (54%)	27 (57.4%)	74 (55.2%)	0.720
Yes	40 (46%)	20 (42.6%)	60 (44.8%)	
Ascites ml	1364 ± 1431	1425 ± 1620	1377 ± 1466	0.913
Volume of fluids intraoperatively ml, median (IQR)	5500 (3500–7280)	4500 (3000–6297)	5420 (3500–6947)	***0.048***
Crystalloids ml, median (IQR)	4000 (3000–5000)	3500 (2500–4500)	4000 (3000–5000)	***0.036***
Colloids ml, media (IQR)	1000 (250–1500)	500 (0–1000)	1000 (0–1500)	0.218
Blood products ml, median (IQR)	0 (0–1000)	0 (0–1100)	0 (0–1000)	0.761

Tumor stage: based on the FIGO, International Federation of Gynecology and Obstetrics [20]; Interval, surgery after primary Chemotherapy

*CRS* cytoreduction surgery; *LOS* length of stay; *RD* residual disease; *SD* standard deviation; *HE* Hysterectomy; *BSE* bilateral Salpingovarectomy; *LNE* Lymphonodectomy

### Postoperative management and postoperative complications

Data on postoperative management is given in Table 3. Outcome data and data on postoperative complications are listed in Table 4. Significantly less patients in the ERAS group suffered complications (ERAS group: 14 (29.8%) vs. pre-ERAS: 46 (52.9%); *p* = 0.011). Total LOS was significantly shorter in the ERAS group (ERAS: 11 (9–12) vs pre-ERAS: 13 (11–16) days; *p* < 0.001)). LOS on ICU did not differ in both groups. However, in the ERAS group, 12.8% of patients were not admitted to ICU at all, whereas this fraction was only 2.3% in the pre-ERAS group (*p* = 0.022). Patients in the ERAS group were mobilized out of bed relevantly earlier (ERAS group: 48.9% vs pre-ERAS: 35.6%, *p* < 0.001). In particular, the number of urinary tract infections (ERAS group: 3 (6.3%) vs. pre-ERAS group: 17 (19.5%); *p* = 0.045), and of paralytic Ileus (ERAS group: 0 (0%) vs. pre-ERAS group: 10 (11.5%); *p* = 0.015) was considerably lower.

**Table 3:** Pre- and postoperative management

Characteristics	Pre-ERAS-group *n* = 87	ERAS-group *n* = 47	Total *n* = 134	*P*
Preoperative education	0 (0%)	42 (89.4%)	42 (31.3%)	** < *0.001***
PONV-prophylaxis (*n*. %)	29 (33.3%)	27 (57.4%)	56 (41.8%)	***0.014***
Carbohydrate loading (*n*. %)	5 (5.7%)	42 (89.4%)	47 (35.1%)	** < *0.001***
Epidural catheter (*n*. %)	69 (79.3%)	36 (76.6%)	105 (78.4%)	0.696
Preoperative thrombosis prophylaxis	34 (39.1%)	0 (0%)	34 (25.4%)	** < *0.001***
Length of stay ICU				
0 night	2 (2.3%)	6 (12.8%)	8 (6.0%)	***0.022***
1 nights	64 (73.6%)	29 (61.7%)	93 (69.4%)	
2 nights	18 (20.7%)	9 (19.1%)	27 (20.1%)	
3 nights	3 (3.4%)	2 (4.3%)	5 (3.7%)	
7 nights	0 (0%)	1 (2.1%)	1 (0.7%)	
Time to termination of urinary drainage (nights) median (IQR)	3 (2–5)	2 (2–4)	3 (2–4)	***0.003***
Time to termination of EC (nights)	4 (1.5–5.5)	3 (1–5)	4 (1–5)	0.119
Time pain control with oral analgetics (nights)	6 (5–8)	5 (3.5–6)	6 (4–7)	** < *0.001***
Use strong opioids postoperative 48 h (*n*. %)	48 (55.1%)	17 (36.2%)	65 (48.5%)	***0.002***
Use NSAIDS postoperative (*n*. %)	18 (23.4%)	15 (31.9%)	33 (26.8%)	0.304
Duration of mobilization per day				
DOS *n* (%) non moved	56 (68.3%)	24 (77.4%)	80 (59.7%)	***0.011***
POD 1 (min) median (IQR)	10 (0–20)	15 (0–30)	10 (0–20)	** < *0.001***
POD 2 (min) median (IQR)	20 (10–22.50)	30 (15–48.75)	20 (10–30)	***0.030***
POD 3 (min) median (IQR)	30 (15–45)	45 (20–67.50)	30 (16.25–60)	0.155

*ICU* intensive care unit; *EC* epidural catheter; *NSAID* non-steroidal anti-inflammatory drugs; *DOS* day of surgery; *POD* postoperative day

**Table 4:** Outcome and complications

Characteristics	Pre-ERAS-group *n* = 87	ERAS-group *n* = 47	Total *n* = 134	*P*
Residual disease (*n*. %)				
None	47 (54%)	27 (57.4%)	74 (55.2%)	0.720
Yes	40 (46%)	20 (42.6%)	60 (44.8%)	
Length of hospital stay (days) Median (IQR)	13 (11–16)	11 (9–12)	12 (10–14)	** < *0.001***
Discharged within 30 postoperative days (*n*. %)	84 (95.5%)	47 (100%)	131 (97.8%)	0.709
30-days mortality (*n*. %)	3 (3.4%)	0 (0%)	3 (2.2%)	0.552
Patients with postoperative complications (*n*. %)	46 (52.9%)	14 (29.8%)	60 (44.8%)	***0.011***
Grade of complications (26)				
I-IIIA	37 (42.5%)	12 (25.5%)	49 (36.6%)	0.132
IIIB-V	9 (10.5%)	2 (4.3%)	11 (8.2%)	0.590
Infections				
Pneumonia (*n*. %)	6 (6.9%)	1 (1.2%)	7 (5.2%)	0.421
Wound infection (*n*. %)	13 (14.9%)	3 (6.4%)	16 (11.9%)	0.173
Urinary tract infection (*n*. %)	17 (19.5%)	3 (6.3%)	20 (14.9%)	***0.045***
Abdominal abscess (*n*. %)	5 (5.7%)	0 (0%)	5 (3.7%)	0.162
Sepsis (*n*. %)	4 (4.6%)	0 (0%)	4 (3%)	0.297
Patients with at least one infection (*n*. %)	29 (33.3%)	6(12.8%)	35 (26.1%)	***0.013***
Cardiovascular events				
DVT (*n*. %)	1 (1.1%)	0 (0%)	1 (0.7%)	1.000
Pulmonary embolism (*n*. %)	4 (4.6%)	4 (8.5%)	8 (6%)	0.450
Cardiac events (*n*. %)	4 (4.6%)	1 (1.2%)	5 (3.7%)	0.657
Patients with at least one cardiovascular complication (*n*. %)	8 (9.2%)	5 (10.6%)	13 (9.7%)	0.788
Renal complications (*n*. %)	2 (2.2%)	1 (2.1%)	3 (2.2%)	0.633
GI complications				
Anastomotic leaks (*n*. %)	3 (3.4%)	0 (0%)	3 (2.2%)	0.701
Nausea or vomiting (*n*. %)	12 (13.8%)	2 (4.3%)	14 (10.4%)	0.137
Ileus mechanical (*n*. %)	4 (4.6%)	1 (2.1%)	5 (3.7%)	0.657
Ileus paralytic (*n*. %)	10 (11.5%)	0 (0%)	10 (7.5%)	***0.015***
Patients with at least one GI complication (*n*. %)	18 (20.7%)	2 (4.3%)	20 (14.9%)	** < *0.001***
Hospital readmission (*n*. %)	18 (20.7%)	7 (14.9%)	25 (18.7%)	0.49

*RD* residual disease; Grade of postoperative complication [21]; *DVT* deep vein thrombosis; *PE* pulmonary embolism, *GI* gastrointestinal

## Discussion

The results of this study give evidence that implementation of an ERAS protocol for patients undergoing extended cytoreductive surgery for ovarian cancer leads to a reduction of postoperative complications and a reduction of LOS in hospital. In contrast to earlier investigations by Bisch et al. [22] and Dickson et al. [23], we studied exclusively patients with ovarian cancer. However, despite this higher-risk population, we observed a comparable, highly significant reduction of LOS in hospital of 2 days.

Since 2015, single Fast-Track elements, such as renunciation of routine bowel preparation, fluid restriction, early start of food intake and mobilization, early removal of urinary drainage, and the use of epidural catheters had been implemented into our ovarian cancer surgery program. However, those aspects, although recommended, were not obligatorily established. The here presented comparison of the ERAS group with the historical control group (pre-ERAS) from 2015 to 2017 strengthens the hypothesis that a consistent implementation and strict adherence to an ERAS protocol bundling all single components, is a further step toward improvement of outcome in this patient population.

Additional aspects that were added to our patient management, and most importantly that were also clearly compulsory regulated by written standard operating procedures, comprised extended preoperative patient education on ERAS, the avoidance of preoperative bowel preparation, the reduction of preoperative fasting time, preoperative metabolic optimization by carbohydrate loading, early postoperative enteral feeding, avoidance or early abandoning of wound drains and feeding tubes, strict peri- and postoperative pain therapy using epidural anesthesia and opioid sparing medication, and early mobilization out of bed after surgery.

Historically, the ERAS protocol for ovarian cancer surgery has been developed on the basis of the protocol for colorectal surgery. It is common sense that in colorectal surgery, the most important singular aspect for beneficial effects is the use of laparoscopy as surgical technique leading to a significant reduction of the surgical trauma [24–26]. This advantage, also in particular combination with adapted ERAS protocols, could also be transferred to gynecological low-complex surgical procedures such as elective, non-oncological hysterectomy [27]. This major aspect, the consequent use of laparoscopy is not possible in extended cytoreductive surgery for ovarian cancer. Therefore, it is highly remarkable, that here the implementation of an ERAS protocol had such significant impact on outcome, even though POSSUM score indicated that patients of the ERAS group had an elevated perioperative risk for complications compared to the pre-ERAS group.

A limitation of our study is the comparison to a historical control group. Even if the baseline variables are comparable, it cannot be excluded that differences regarding the outcome are caused by time effects regarding the treatment strategies. However, our data are in many aspects well comparable to the recently published PROFAST study [28]. In this randomized controlled, prospective study, Sánchez-Iglesias et al. could impressively show that the implementation of ERAS in this patient group led to a reduction in hospital stay of 2 days, and in hospital readmission rate of 14%. Our data confirm these findings. Patient collectives in both groups were widely comparable with regard to preoperative findings, surgical invasiveness, and histopathological findings. Also regarding age, patients in the non-ERAS groups of both studies were identical. Interestingly, the patients of the ERAS group in our study were nearly 8 years older. This might point towards a beneficial effect of ERAS specifically in older patients. With regard to surgical radicalness, it is striking that patients of the ERAS group received considerably less frequent a systemic radical lymphonodectomy (31.9% (ERAS group) vs. 64.4% (pre-ERAS group); *p* < 0.001). This change in clinical practice was grounded on the findings of the LION study, which demonstrated less postoperative complications, when avoiding this surgical procedure [29]. Further, by trend, in less patients of the ERAS group surgery was extended to the upper abdomen (78.7% (ERAS group) vs. 92.90% (pre-ERAS group); *p* = 0.054). Both of these factors might have contributed to the reduced complication rate of the ERAS group. On the other hand, patients in the ERAS group had a higher POSSUM score (11.9 (ERAS group) vs. 9.4 (pre-ERAS group); *p* < 0.001), reflecting a relevantly higher surgical risk. It is remarkable that even so, less patients of the ERAS group needed postoperative ICU admission (12.8% (ERAS group) vs. 2.3% (pre-ERAS group); *p* = 0.022).

Further, the significantly lower incidence of gastrointestinal complications, and here in particular of paralytical ileus in the ERAS group (11.5% vs 0% *p* = 0.015) is remarkable. This is in contrast to a recent data analysis by Kalogera et al., who reported less nausea in the ERAS cohort, however, no reduction in the occurrence of paralytical ileus [30]. We interpret that effect as a combined consequence of faster postoperative mobilization, earlier enteral feeding, postoperative use of chewing gum, and the reduced use of strong opioids within the first 48 h post-surgery (55.1% (pre-ERAS group) vs. 36.2% (ERAS group); *p* = 0.002). Also, the pre-surgery use of carbohydrate loading (5.7% (pre-ERAS group) vs. 89.4% (ERAS group); *p* < 0.001), and the increased use of PONV prophylaxis, (33.3% (ERAS group) vs. 57.4% (pre-ERAS group); *p* = 0.014) might have contributed here. It is a frequently cited clinical notion that epidural anesthesia contributes to prolonged postoperative gut dysfunction [31]. It is noteworthy that in our study improved postoperative gut function could be observed even though epidural anesthesia was used in the same extent as in the pre-ERAS group. Much more, it is our clinical experience that a consistent use of epidural anesthesia in the postoperative phase facilitates early mobilization, which is another key feature of ERAS. In our study, patients of the ERAS group had longer time periods of mobilization within the first 3 days (see Table 3, *p* < 0.001). Additionally, we confirm the report by Bergstrom and colleagues regarding postoperative pain [32]: also in our exclusively oncological patient cohort, pain control was achieved earlier in the ERAS group (time to pain control with oral analgesics: 5 (ERAS group) vs. 6 (pre-ERAS Group); *p* < 0.001), and this even with a reduced fraction of patients with the need of strong opioids (36.2% (ERAS group) vs. 55.1% (pre-ERAS group; *p* = 0.002). We speculate that early mobilization, which is only possible with an optimized pain therapy, is one main factor for that reduction in postoperative gastrointestinal dysfunction.

The ERAS protocol demands early removal of any kind of drains: this effect can be clearly recognized in our data, as the use of Foley catheters was significantly shorter in the ERAS group (2 (ERAS group) vs. 3 nights (pre-ERAS group); *p* = 0.003). The clinical effect is reflected in the reduced number of urinary tract infections in patients of the ERAS group (3 (6.3%) (ERAS group) vs. 17 (19.5%) (pre-ERAS group; *p* = 0.045).

After analysis of our data, we became skeptical concerning the non-existing recommendation for pre-operative pharmacological thrombosis prophylaxis in the current ERAS protocol. Following this protocol, our patients did not receive preoperative anticoagulation, whereas this was performed in 39.1% of patients in the pre-ERAS group. We recognized a signal of increased occurrence of pulmonary embolisms in the ERAS group (8.5% (ERAS group) vs. 4.6% (pre-ERAS group); *p* = 0.450) despite a 100% compliance of postoperative anticoagulation in both groups. Since analysis of our data, we changed our clinical practice, and we now give preoperative low molecular heparin in prophylactic dosage on a routine basis.

Muallem et al. observed that the implementation of ERAS elements in gynecologic oncology in Germany is still not satisfying as only half of the departments are nowadays able to apply at least 70% of the ERAS elements. We agree with their conclusion that it is of high clinical and scientific relevance to consistently build up a structured database for gynecological oncology units on national and international level to facilitate and monitor further implementation and standardization of the ERAS protocol [33].

In conclusion, our data strongly support the use of a consistent multimodal ERAS treatment program in patients undergoing cytoreductive surgery for ovarian cancer. It led to a reduction of postoperative morbidity, and in particular to a reduced rate of infectious and gastrointestinal complications, which allowed earlier discharge from hospital. These patients generally require subsequent oncological chemotherapy after surgery. Since there is evidence that the timespan between surgery and initiation of subsequent chemotherapy is relevant for long-term survival [16], ERAS might therefore even indirectly contribute to better long-term outcome. However, this needs to be investigated by further studies.

## Data Availability

Data will be shared on well-founded request.
